# Stability of R2* and quantitative susceptibility mapping of the brain tissue in a large scale multi-center study

**DOI:** 10.1038/srep45261

**Published:** 2017-03-28

**Authors:** Rongpin Wang, Guangyou Xie, Maoxiong Zhai, Zhongping Zhang, Bing Wu, Dandan Zheng, Nan Hong, Tao Jiang, Baohong Wen, Jingliang Cheng

**Affiliations:** 1 Department of Radiology, Guizhou People’s Hospital, Guiyang, 550002, China; 2 GE Healthcare MR Research China, Beijing, 100176, China; 3 Department of Radiology, Beijing Univ. People’s Hospital, Beijing, China; 4 Department of Radiology, Beijing Chaoyang Hospital, Beijing, China; 5 Department of Radiology, Zhengzhou Univ. 1st Hospital, Zhengzhou, China

## Abstract

Multi-center studies are advantageous for enrolling participants of varying pathological and demographical conditions, and especially in neurological studies. Hence stability of the obtained quantitative R2* and susceptibility in multicenter studies is a key issue for their widespread applications. In this work, the stabilities of simultaneously obtained R2* and susceptibility are investigated and compared across 10 sites that are equipped with the same scanner and receiver coil, the same post-processing process was used to achieve consistent experiment setup. Two healthy adult volunteers (one male and female) participated in this study. High intraclass correlation coefficient was obtained for both susceptibility (0.94) and R2* (0.96). The coefficients of variance for all measurements obtained were smaller than 0.1, the largest variations of measurements in all the chosen ROIs fall within ±20% from the median value. Higher level of stability was obtained in R2* as compared to susceptibility at 1 mm resolution (P < 0.05) and at 1.5 mm (P < 0.01).

Magnetic resonance imaging is known and well accepted for its non-invasiveness and rich of image contrasts. Anatomical MR imaging are often based on the weighting of varying spin relaxation rates of different body tissues under a static magnet field and the mutual act of radio frequency pulses. However the obtained MR images are not directly quantitative due to the different imaging conditions of different scanners at different time points, even the identical imaging sequence is used. As a result, the interpretation of MR images is still based on qualitative inspection of hyper-intensity or hypo-intensity as compared to the surrounding tissues.

The search for novel and quantitative imaging contrast is an ever-existing driving force in the MR research community. This desire is especially strong in neuro-imaging community due to brain’s complex structure and composition. In the studies of brain functions and neurodegenerative diseases, often no or very little morphological changes are present. BOLD imaging^1^, resting state imaging^2^, diffusion weighted imaging^3^ and perfusion weighted imaging^4^ have been used to reveal brain functions related signal intensity changes. Another interesting and useful aspect in imaging is to capture the brain tissues’ composition changes, which may take place prior to the morphological and functional changes.

Many substances in the brain’s chemical compositions affect its functioning and are indicators of neurological disorders. However, imaging of these substances is usually difficult due to their very limited presence. One means for their detection is to exploit the fact that they often resonate at different frequencies than that of water, that may cause the local field shift and inhomogeneity, T2* (R2*) and quantitative susceptibility^5^ are direct consequences of this. As has been shown, the R2* and susceptibility contrast in the white matter and grey matter are mainly contributed by the myelin and iron respectively, which are important biomarker for studying the brain’s development and neurological disorders. They have been applied in a variety of studies including ageing^6^,^7^,^8^, functional study^9^, perfusion^10^, Parkinson disease^11^, Alzheimer disease^12^, calcification^13^, multiple sclerosis^14^,^15^, etc.

Multi-center studies are advantageous for involving participant of varying pathological and demographical features, and becoming popular in neurological studies. Hence the stability of the obtained quantitative R2* and susceptibility is a key issue for their widespread applications, given the fact that many factors may introduce variance to the final measurements. There have been recent attempts in investigating this issue: Lin *et al*.^16^ first investigated the longitudinal reproducibility on three different scanners; Hinoda *et al*.^17^ reported the data consistency and reproducibility at 1.5 T and 3.0 T; Deh *et al*.^18^ further refined on the data processing technique and also included two field strengths; Santin *et al*.^19^ reported on the effects of various processing methods on the resulting measurements on the same scanner. However, only several scanners with different data acquisition platforms from one or two sites were included in previous studies, this may limit the assessment on the inter site reliability as different image acquisitions and hardware configurations may introduce bias into the resulting measurements.

In this study, the reproducibility of simultaneously obtained R2* and susceptibility of the same subjects is investigated across 10 sites that are equipped with the same scanner and receiver coil. The derivation of susceptibility map was then performed with the same post-processing software. The goal is to investigate what level of stability we may expect in a large scale multi-center study, if consistency of the experimental setup is maintained.

## Results

### Image contrast generated

**Figure 1:**
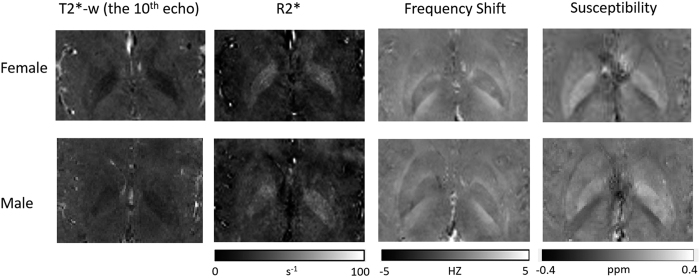
Axial slices of T2* weighted magnitude image (10^th^ echo), R2* map, frequency shift map and susceptibility map derived from the multi-echo GRE sequence for the male and female subjects.

**Figure 2:**
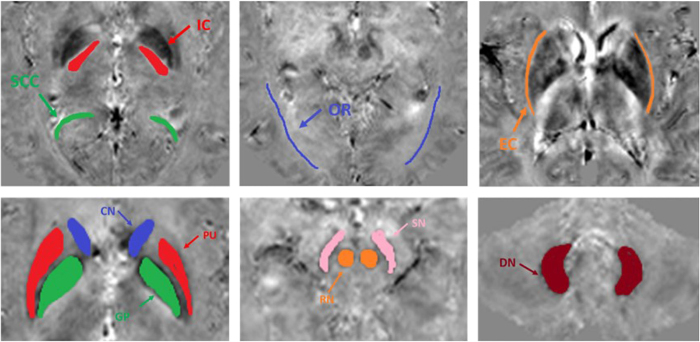
ROIs were defined and delineated based on the susceptibility maps (shown as subject 1): SCC, IC, OR, EC in the white matter (top row); GP, CN, PU, RN, SN, DN in the grey matter (bottom row).

The magnitude images from the 10th echo, which was used for image registration, the echo-combined frequency shift map, the R2* map and the susceptibility map derived are shown in Fig. 1. The latter three images display better SNR as they were derived from data at different echo times. It is seen that all the images delineate white matter and grey matter, but the contrast is relatively weak on the magnitude and R2* images as compared to frequency shift and susceptibility maps. It is also seen that the boundaries of the deep nuclei regions are less definitive on the frequency shift maps as compared to the susceptibility maps due to the local dipole effects. These observations are consistent with previous reports. Also the R2* and susceptibility maps allow for direct quantification, as indicated by the scale bar. Illustrations of the ROI selections on white matter and grey matter for subject 1 are shown in Fig. 2, the actual ROIs were volumetric whereas only representative slices were shown.

### Statistical analysis

**Figure 3:**
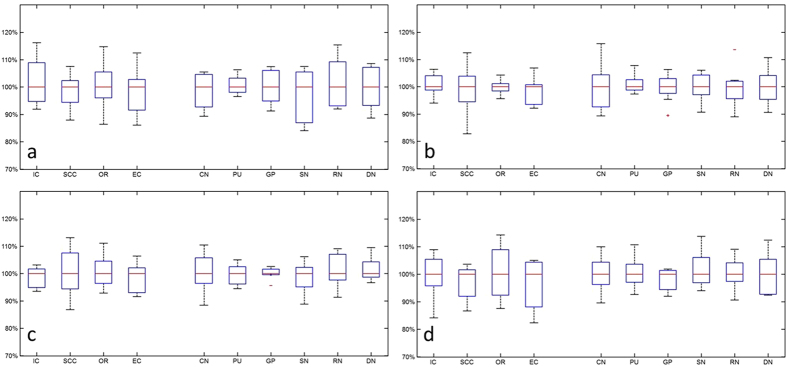
Box whisker plots of median normalized measurements of (**a**,**c**) susceptibility and (**b**,**d**) R2* in (**a**,**b**) male subject and (**c**,**d**) female subject at 1 mm resolution. The ROIs in white matter and grey matters are as labeled and separated by white space.

**Figure 4:**
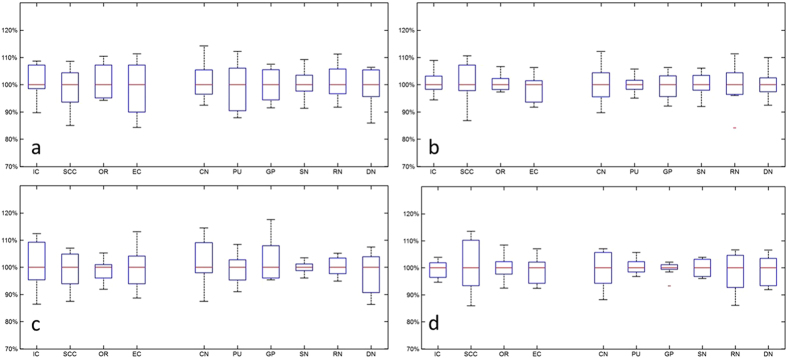
Box whisker plots of median normalized measurements of (**a**,**c**) susceptibility and (**b**,**d**) R2* in (**a**,**b**) male subject and (**c**,**d**) female subject at 1.5 mm resolution. The ROIs in white matter and grey matters are as labeled and separated by white space.

**Figure 5:**
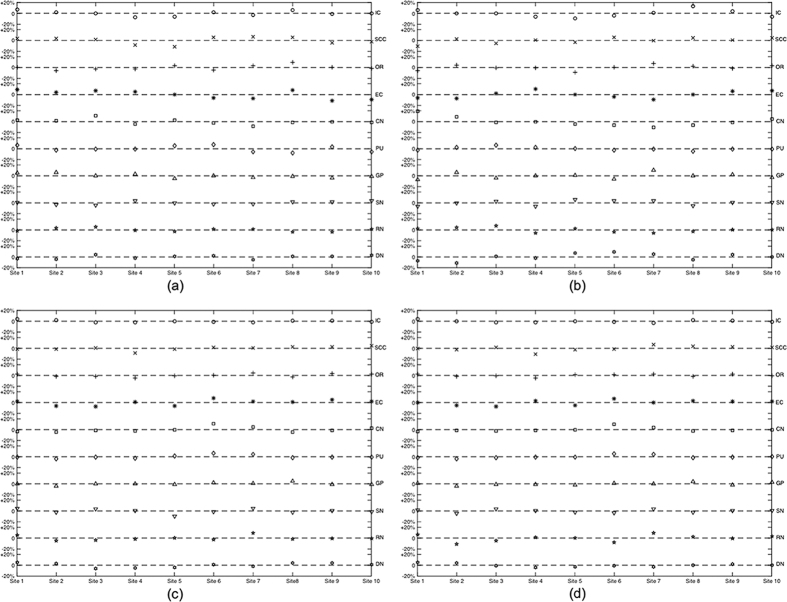
Plots showing the deviations levels of the individual ROI and site measurements from the site averaged measurement for (**a**) susceptibility at 1 mm; (**b**) susceptibility at 1.5 mm; (**c**) R2* at 1 mm; (**d**) R2* at 1.5 mm. The shift from the mean (solid line) represents the percentage of deviations, and different sites and ROIs are as labeled.

The calculated intraclass correlation coefficients (ICCs) and coefficients of variation (CVs) are summarized in Table 1. It is seen the median ICC (ranged from 0.94 to 0.99) values as well as the corresponding 95% CIs indicate high reproducibility for both susceptibility and R2*. Varying levels of CVs were obtained for different ROIs, and the overall CV was low for all ROIs (<0.1). The level of measurement variance may be better visualized in Figs 3 and 4. In the box-whisker plots of median-normalized measurements, the extents of the box and whisker directly indicate the percentage of the variation from the median value and hence show the distribution of the variances. It is seen that the variance of all measurements from different sites, as indicated by the extent of the two whiskers, fall within ±20% from the median value, including two outliers that are outside 99.3% data percentile as indicated by the red markers; in many cases, the variances of the measurements were constrained within ±10% from the median value. This is consistent with the CV levels in Table 1 (<0.1). Statistically significant difference was observed between the CVs of susceptibility and R2*, and between measurements at different resolutions, as revealed by the paired t test as summarized in Table 2. No difference was observed between the CV of susceptibility at 1.5 mm and R2* at 1 mm. As shown by the results, R2* featured a better level of stability as compared to susceptibility at both 1 mm (p < 0.05) and 1.5 mm (p < 0.01). Figure 5 reveals the cross-site-mean normalized measurement derivations (shown in percentage) of each site and ROI (measurements were averaged over the two subjects). From the plot, it can be seen that no site showed systematical errors for all the four metrics.

### Correlation of R2* and susceptibility in deep nuclei regions

**Figure 6:**
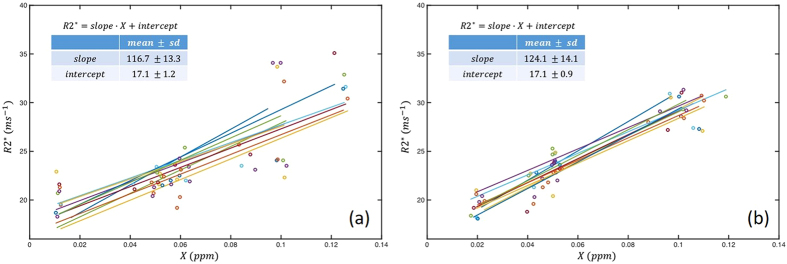
Scatter plot superimposed with linear regressions between R2* and susceptibility measurements in deep nuclei regions from different sites of (**a**) male and (**b**) female subject at 1 mm resolution. The mean and standard derivations of the fitted slope and intercepts are shown in the right corner of the plots.

**Figure 7:**
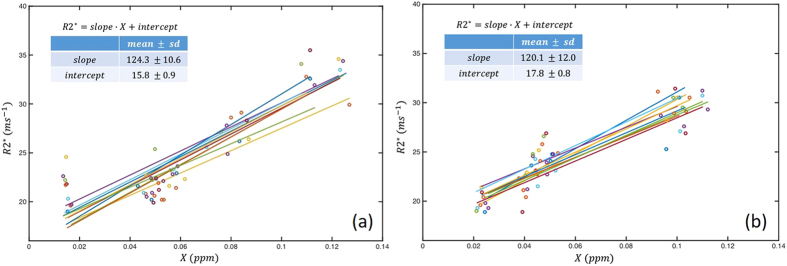
Scatter plot superimposed with linear regressions between R2* and susceptibility measurements in deep nuclei regions from different sites of (**a**) male and (**b**) female subject at 1.5 mm resolution. The mean and standard derivations of the fitted slope and intercepts are shown in the right corner of the plots.

Figures 6 and 7 show the scatter plot and line fitting of R2* and susceptibility values in deep nuclei regions at 1 mm and 1.5 mm resolution respectively. In general, strong positive linear correlations were observed, and visually the resulting linear fitting were similar among measurements from different sites. The variations of the fitting were assessed by the mean and standard derivations of the slopes and intercepts of the resulting linear fitting as shown in the top left corner of the plots. The level of variance of the fitted linear model is in similar range to those of the direct measurements, and similar level of variance of the fitted linear model is observed in the two subjects.

## Discussion

In this study, by keeping all the controllable experiment setup and post-processing steps consistent, the stabilities of R2* and susceptibility across different sites were investigated. As compared to previous work^16^,^17^,^18^,^19^, the main goal of this study is to see what level of variance in R2* and susceptibility measurements should be expected and tolerated given other inevitable influencing factors, as if in the cases of a large scale multi-center study.

Two subjects participated in this study and measurements were obtained across 10 sites. High reliability was observed in the susceptibility and R2* measurements. This is consistent with previous reports, and smaller levels of variance were obtained: the intra-subject variation as measured by coefficient of variance was constrained to be within 0.1; and the largest variations of measurements in all the chosen ROIs fall within ±20% from the median value, which translate to about ±20 *ppb* for GP and as little as ±4 *ppb* for CN. The considerably smaller level of variance as compared to previous studies is attributed to the consistent imaging setup across all the sites. The level of stability varied among different ROIs and subjects, as attributed to different chemical compositions and subjects’ pathological conditions^6^.

Provided identical hardware and software setup, there are still factors that may contribute to the variations of the final measurements: real time B0 field homogeneity, gradient eddy current, real time setting of central frequency, subjects’ head orientation, noise and imperfect image registration, etc. These are the factors that cannot be completely avoided if multi-center data acquisition is to be conducted in clinical practice. But attention could be paid to minimize the effects as practiced in this study. Which of these factors is more dominant remains a question that cannot be well answered in this study. Subjects’ orientation is a unique factor in this experiment. As shown in refs 20, 21, 22, R2* and susceptibility in both white and grey matter are anisotropic and their numeric values are dependent on the object orientation to the main field. In this study, the subjects’ heads were attempted to be kept in the same position across different imaging sessions, and the subjects were very cooperative. Hence little contribution should be expected from the orientation difference.

Noise is often considered as a main player in the variance in repeated measurements. In this study, high ICC indicated that most of the observed variance was real and may be attributed to noise. Retrospectively down-sampled data was used to assess the convolved effects of SNR improvement and lowered resolution. The latter may affect the outcome of the image registration and the increased level of partial volume effects may also affect the measurement results. However, the image resolution itself has been reported not to be an influential factor to the measured susceptibility values^23^. Overall, the SNR benefit outweigh the resolution effects and improved stability was observed in both R2* and susceptibility with a resolution of 1.5 mm. In addition, the stability of R2* were seen to benefit more from the increased SNR, this maybe because the multi-echo combined frequency shift has sufficiently high SNR, and the additional SNR improvement became marginal.

It was interesting to observe that R2* showed better stability than susceptibility across different sites, as revealed by the paired t test of the CVs of different ROIs. This observation is consistent with that reported by Santin *et al*.^19^. Two potential factors may cause this: firstly, the susceptibility reflects the local field shift from the assumed water center frequency, which is more venerable to subtle difference of the real time B0 field and shimming; secondly, the derivation of susceptibility involved non-deterministic steps, which may amplify the noise propagation into the final reconstruction.

There are several limitations in this study. Firstly, the number of participants was limited due to logistic difficulty. As a result, measurements of different brain regions and measurements at different resolutions were treated as the within group subjects in the reliability test. With a larger number of participants and multiple scan sessions, it would be possible to look at the levels of reproducibility for measures of different brain regions with statistical power. This could be an interesting aspect to investigate. The limited number of subjects may also introduce measurement variations related to subject preparations in each site, which may impact the study outcome. Secondly, the derivations of susceptibility involve several steps of non-deterministic calculation and each step may affect the propagations of errors and noise. As reported by Santin *et al*.^19^, different methods do lead to varying levels of stabilities. Hence the level of stabilities obtained in this study is associated with this particular reconstruction method. Furthermore, the QSM derivation algorithm adopted assumes a zero reference that may impact the iteration outcome and was not taken into consideration in this work. Such offset maybe eliminated by involving a reference susceptibility such as that of cerebral spinal fluid (CSF) in the final calculation.

## Conclusion

High level of stability of both R2* and susceptibility may be expected in multi-center studies, if consistency in experimental setup is maintained. Different brain regions feature varying level of stabilities, and R2* features a higher level of stability than susceptibility.

## Methods

### Experiment design

This prospective study was approved by the Ethics Committee of Guizhou People’s Hospital and written informed consents were obtained from all volunteers. This study was conducted in accordance with the Declaration of Helsinki. Two healthy adult volunteers (one male and one female, age of 31 and 29) participated in this study as subjects. Consent forms were obtained from the volunteer and approvals of scan were also obtained at local sites where scans were performed. MRI scans were performed at 10 different sites installed with Discovery MR750 (General Electric, Milwaukee) scanners equipped with an 8-channel head coil (*In*-*vivo*, Florida). The sites and scan times were: 1. Peking university people’s hospital (07/13/2015, 12 am); 2. Cancer hospital Chinese academy of medical sciences (07/20/2015, 2 pm); 3. Beijing hospital (07/20/2015, 9 pm); 4. Tsinghua Changgung hospital (07/20/2015, 11 pm); 5. Institute of psychology, CAS (07/21/2015, 11 am); 6. China-Japan friendship hospital (07/21/2015, 1 pm); 7. PLA 301 hospital (07/21/2015, 2 pm); 5. Peking University (07/31/2015, 5 pm); 9. Guizhou people’s hospital (08/03/2015, 4 pm); 10. Zhengzhou university first hospital (08/05/2015, 11 am). The same MR protocol were scanned and consisted of three sequences: 1) Localizer, 2) calibration scan, 3) 3D multi-echo SPGR. The imaging parameter of the multi-echo GRE sequence is as following: FOV = 220 mm, TR = 29.7 ms, 12 echoes, first TE = 1.94 ms, echo spacing = 2.1 ms, read-out bandwidth = ±62.5 kHz, acquisition matrix = 220 × 220, slice thickness = 1 mm. Full brain coverage was achieved with a total slice number of 140. Bipolar echo readout was used. An acceleration factor of 2 in the phase encoding direction was used that led to a total scan time of 4:57 minutes. All scanners were in normal functioning order and passed 2^nd^ order shimming. Automatic coarse and fine adjustment of the central frequency based on variable flip angle tests as well as linear gradient shimming were performed prior to the multi-echo GRE acquisition. The subjects were instructed to keep head untilted along the S/I direction and still during the scan. The same landmark beneath the noise was used.

### Image reconstruction

Parallel imaging reconstruction was first performed to restore the images at different echo times. Both magnitude and phase images were obtained. R2* maps were calculated by performing a voxel-by-voxel mono-exponential fitting using all the magnitude images at 12 echo times. Susceptibility maps were derived from the phase images at different echo times with the following procedure: 1) Laplacian-based phase unwrapping was performed on phase image at each echo time^18^; 2) The unwrapped phase maps from different echo times then normalized by the corresponding echo times and averaged to yield the frequency shift; 3) SHARP background phase removal^24^ with a varying diameter^18^,^25^,^26^ was performed on the frequency map at each echo times; 3) frequency shifts at different echo times are combined as weighted by their echo times^27^,^28^; 4) derivation of the susceptibility map from the combined frequency shift map was performed using the conditioned LSQR method^18^. The derivation of the susceptibility map was performed using the STI suite toolbox as available online at http://people.duke.edu/~cl160/index.html, a stopping criteria of lamda <0.01 was used in the computation of all the susceptibility data sets and convergence was achieved in each case.

### Data measurements

Analysis of the reconstructed R2* and susceptibility maps follow the following three- step procedure: 1) all the images were registered to common template using the FLIRT method in FSL software^29^ based on the magnitude image from the 10^th^ echo; 2) multi-slice ROIs were manually placed on the various white matter and grey matter regions on one susceptibility map data set, and copied to other image sets; 3) the averaged values in the ROIs are used as the measurement for comparisons. The selected ROIs included: internal capsule (IC), splenium of corpus callosum (SCC), and optic radiation (OR) in the white matter; putamen (PU), globus pallidus (GP), caudate nuclei (CN), red nuclei (RN), substantia nigra (SN), and dentate nuclei (DN) in the grey matter. The ROIs were defined to cover the entire volume of the selected anatomical regions, these regions were selected as they have been previously reported to have dominant contribution of R2* and frequency shifts of myelin and iron respectively. Linear fittings were performed for R2* and susceptibility measurements in white matter and grey matter respectively.

Since the data acquisition at 1 × 1 × 1 mm resolution requires nearly 5 minutes and poses an issue on the acquisition time, considering both the scan throughput as well as the vulnerability to motion. All the datasets were retrospectively down-sampled to approximately 1.5 mm in k-space by discarding the high frequency components at the edge of the 3D k-space (from 220 × 220 × 136 to 146 × 146 × 94), which reflected a scan time of just 2:12. There are two consequences with down-sampling. Firstly, the down-sampled data set would feature an improved intrinsic SNR. Secondly, down-sampled data set features increased level of partial volume effects, which may the outcome of both measures. The same reconstruction was performed on the down sampled data sets.

### Statistical analysis

Statistical analysis was performed using SPSS version 23. There are two aspects of the statistical analysis: firstly, to assess the reliability of the susceptibility and R2* measures; secondly, to assess if susceptibility or R2* features a higher level of stability. The absolute reliability was calculated using two way random intraclass correlation coefficient (ICC) which reflects the portion of the real changes to the total variation in the measurements. Median ICC and the 95% confidence interval were calculated over all the ROIs and both subjects. The coefficient of variance (CV)^19^ across both subjects was calculated for each ROI, and paired t test was performed to determine if there is a difference in the stabilities of susceptibility and R2* in each ROI. A P value < 0.05 was considered statistically significant. Normalized box-whisker plots were also made for each measurement, so that the variance as indicated by the distance of the ‘box’ and ‘whisker’ from the median level may be directly visualized. The derivations of the measurements in each site from the mean measurement across all the sites were also calculated to investigate if there exists any systematical bias in a particular site. Furthermore, linear regression was performed between R2* and susceptibility measured in the deep nuclei regions, and the resulting slopes and intercepts are compared.

## Additional Information

**How to cite this article:** Wang, R. *et al*. Stability of R2* and quantitative susceptibility mapping of the brain tissue in a large scale multi-center study. *Sci. Rep.* **7**, 45261; doi: 10.1038/srep45261 (2017).

**Publisher's note:** Springer Nature remains neutral with regard to jurisdictional claims in published maps and institutional affiliations.

## Figures and Tables

**Table 1:** Intraclass correlation coefficient and coefficient of variances of susceptibility and R2* in different ROIs.

	X 1 mm	R2* 1 mm	X 1.5 mm	R2* 1.5 mm
ICC	0.94	0.98	0.97	0.99
95% CI	0.91, 0.96	0.96, 0.99	0.94, 0.99	0.98, 0.99
	**Coefficient of variance**			
IC	0.0652	0.0488	0.0570	0.0417
SCC	0.0748	0.0776	0.0656	0.0680
OR	0.0589	0.0339	0.0531	0.0289
EC	0.0941	0.0462	0.0901	0.0468
CN	0.0716	0.0797	0.0736	0.0688
PU	0.0907	0.0335	0.0806	0.0326
GP	0.0635	0.0477	0.0618	0.0443
SN	0.0501	0.0435	0.0515	0.0420
RN	0.0605	0.0692	0.0625	0.0542
DN	0.0665	0.0623	0.0601	0.0509

**Table 2:** Paired t test of the stabilities of susceptibility and R2*.

	R2* 1 mm	X 1.5 mm	R2* 1.5 mm
X 1 mm	1 (P < 0.05)	1 (P < 0.03)	1 (P < 0.01)
R2* 1 mm		0 (P = 0.09)	1 (P < 0.02)
X 1.5 mm			1 (P < 0.01)

1 indicates there exists significant difference and 0 indicates there was no significant difference. The P value was also labeled. X represents susceptibility.
